# *svm*PRAT: SVM-based Protein Residue Annotation Toolkit

**DOI:** 10.1186/1471-2105-10-439

**Published:** 2009-12-22

**Authors:** Huzefa Rangwala, Christopher Kauffman, George Karypis

**Affiliations:** 1 Computer Science Department, George Mason University, Fairfax, VA, USA; 2 Bioinformatics Department, George Mason University, Fairfax, VA, USA; 3 Computer Science Department, University of Minnesota, Minneapolis, MN, USA

## Abstract

**Background:**

Over the last decade several prediction methods have been developed for determining the structural and functional properties of individual protein residues using sequence and sequence-derived information. Most of these methods are based on support vector machines as they provide accurate and generalizable prediction models.

**Results:**

We present a general purpose protein residue annotation toolkit (*svm*PRAT) to allow biologists to formulate residue-wise prediction problems. *svm*PRAT formulates the annotation problem as a classification or regression problem using support vector machines. One of the key features of *svm*PRAT is its ease of use in incorporating any user-provided information in the form of feature matrices. For every residue *svm*PRAT captures local information around the reside to create fixed length feature vectors. *svm*PRAT implements accurate and fast kernel functions, and also introduces a flexible window-based encoding scheme that accurately captures signals and pattern for training effective predictive models.

**Conclusions:**

In this work we evaluate *svm*PRAT on several classification and regression problems including disorder prediction, residue-wise contact order estimation, DNA-binding site prediction, and local structure alphabet prediction. *svm*PRAT has also been used for the development of state-of-the-art transmembrane helix prediction method called TOPTMH, and secondary structure prediction method called YASSPP. This toolkit developed provides practitioners an efficient and easy-to-use tool for a wide variety of annotation problems.

*Availability*: http://www.cs.gmu.edu/~mlbio/svmprat

## Background

Experimental methods to determine the structure and function of proteins have been out-paced with the abundance of available sequence data. As such, over the past decade several computational methods have been developed to characterize the structural and functional aspects of proteins from sequence information [1-3].

Support vector machines (SVMs) [4,5] along with other machine learning tools have been extensively used to successfully predict the residue-wise structural or functional properties of proteins [6-10]. The task of assigning every residue with a discrete class label or continuous value is defined as a *residue annotation *problem. Examples of structural annotation problems include secondary structure prediction [8,9,11], local structure prediction [12,13], and contact order prediction [14-16]. Examples of functional annotation problems include prediction of interacting residues [6] (e.g., DNA-binding residues, and ligand-binding residues), solvent accessible surface area estimation [10,17], and disorder prediction [7,18].

We have developed a general purpose protein residue annotation toolkit called *svm*PRAT. This toolkit uses a support vector machine framework and is capable of predicting both a discrete label or a continuous value. To the best of our knowledge *svm*PRAT is the first tool that is designed to allow life science researchers to quickly and efficiently train SVM-based models for annotating protein residues with any desired property. The protocol for training the models, and predicting the residue-wise property is similar in nature to the methods developed for the different residue annotation problems [6-10].

*svm*PRAT can utilize any type of sequence information associated with residues. Features of the residue under consideration, as well as neighboring residues, are encoded as fixed length feature vectors. *svm*PRAT also employs a flexible sequence window encoding scheme that differentially weighs information extracted from neighboring residues based on their distance to the central residue. This flexibility is useful for some problems.

The *svm*PRAT implementation includes standard kernel functions (linear and radial basis functions) along with a second-order exponential kernel function shown to be effective for secondary structure prediction and pairwise local structure prediction [9,19]. The kernel functions implemented are also optimized for speed by utilizing fast vector-based operation routines within the CBLAS library [20]. *svm*PRAT is capable of learning two-level cascaded models that use predictions from the first-level model to train a second-level model. Such two-level models are effective in accounting for the residue properties that are dependent on properties of near-by residues (i.e., the functional or structural property is sequentially autocorrelated). This form of cascaded learning performs well for secondary structure prediction [9,17]. *svm*PRAT is made available as a pre-compiled binary on several different architectures and environments.

In this paper *svm*PRAT has been evaluated on a wide suite of prediction problems, which include solvent accessibility surface area estimation [10,17], local structure alphabet prediction [12,13], transmembrane helix segment prediction [21], DNA-protein interaction sites prediction [6], contact order [15] estimation, and disordered region prediction [7,18]. *svm*PRAT has been used in development of a transmembrane helix orientation prediction method called TOPTMH [22], shown to be one of the best performers on a blind independent benchmark [23]. The *svm*PRAT framework was also used for prediction of ligand-binding sites [24] and was initially prototyped for the YASSPP secondary structure program [9].

Support vector machines are a powerful tool for classification and regression tasks. However, adapting them to the particular case of protein sequence data can be onerous. *svm*PRAT is a tool that allows SVMs to be applied readily to sequence data by automating the encoding process and incorporating a number of different features that are specifically designed for the problem of protein residue annotation.

## Implementation

*svm*PRAT approaches the protein residue annotation problem by utilizing local sequence information (provided by the user) around each residue in a support vector machine (SVM) framework [25,26]. *svm*PRAT uses the classification formulations to address the problem of annotating residues with discrete labels and the regression formulation for continuous values. The *svm*PRAT implementation utilizes the publicly available SVM^*light *^program [27].

*svm*PRAT provides two main programs, one for the learning annotation models (*svm*PRAT-L) and the other for the predicting labels from learned models (*svm*PRAT-P). The *svm*PRAT-L program trains either a classification or regression model for solving the residue annotation problem. For classification problems, *svm*PRAT-L trains one-versus-rest binary classification models. When the number of unique class labels are two (e.g., disorder prediction), *svm*PRAT-L trains only one binary classification model to differentiate between the two classes. When the number of unique class labels are greater than two (e.g., three-state secondary structure prediction), *svm*PRAT-L trains one-versus-rest models for each of the classes i.e., if there are *K *discrete class labels, *svm*PRAT-L trains *K *one-versus-rest classification models. For continuous value estimation problems (e.g., solvent accessible surface area estimation), *svm*PRAT-L trains a single support vector regression (ϵ-SVR) model.

The *svm*PRAT-P program assigns a discrete label or continuous value for each residue of the input sequences using the trained models produced by *svm*PRAT-L. In classification problems, *svm*PRAT-P uses the *K *one-versus-models to predict the likelihood of a residue to be a member of each of the *K *classes. *svm*PRAT-P assigns the residue the label or class which has the highest likelihood value. For regression problems, *svm*PRAT-P estimates a continuous value for each residue.

### Input Information

The input to *svm*PRAT consists of two types of information. Firstly, to train the prediction models true annotations are provided to *svm*PRAT-L. For every input sequence used for training, a separate file is provided. Each line of the file contains an alphanumeric class label or a continuous value i.e., true annotation for every residue of the sequence.

Secondly, *svm*PRAT can accept any general user-supplied features for prediction. For a protein, *svm*PRAT accepts any information as feature matrices. Both, *svm*PRAT-L and *svm*PRAT-P accept these input feature matrices. *svm*PRAT-L uses these feature matrices in conjunction with the true annotation files to learn predictive models, whereas *svm*PRAT-P uses the input feature matrices with a model to make predictions for the residues.

**Figure 1:**
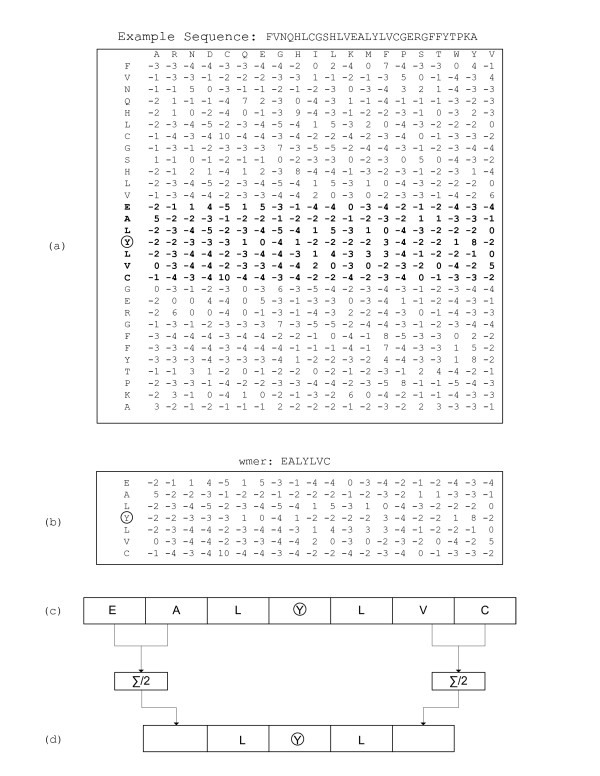
**(a) Input example sequence along with PSI-BLAST profile matrix of dimensions *n *× 20, with a residue circled to show the encoding steps**. (b) Example *w*mer with *w *= 3 giving length seven with extracted features from the original PSI-BLAST matrix. (c) Encoded vector of length 7 × 20 formed by linearizing the sub-matrix. (d) Flexible encoding showing three residues in the center using the finer representation, and two residues flanking the central residues on both sides using a coarser representation as an averaging statistic. Length of this vector equals 5 × 20.

A feature matrix *F *for a protein sequence *X *is of dimensions *n *× *d*, where *n *is the length of the protein sequence and *d *is the number of features or values associated with each position of the sequence. As an example, Figure 1(a) shows the PSI-BLAST derived position specific scoring matrix (PSSM) of dimensions *n *× 20. For every residue, the PSSM captures evolutionary conservation information by providing a score for each of the twenty amino acids. Other examples of feature matrices include the predicted secondary structure matrices and position independent scoring matrices.

We use *F*_*i *_to indicate the *i*th row of matrix *F*, which corresponds to the features associated with the *i*th residue of *X*. *svm*PRAT can accept multiple types of feature matrices per sequence. When multiple types of features are considered, the *l*th feature matrix is specified by *F*^*l*^.

### Information Encoding

When annotating a particular residue, *svm*PRAT uses features of that residue as well as information about neighboring residues. Window encoding, also called *w*mer encoding, is employed to accomplish this. For sequence *X *with length *n*, we use *x*_*i *_to denote the *i*th residue of the sequence. Given a user-supplied width *w*, the *w*mer at position *i *of *X *(*w *<*i *≤ *n *- *w*) is defined to be the (2*w *+ 1)-length subsequence of *X *centered at position *i*. That is, residues immediately before and after *x*_*i *_are part of *w*mer(*x*_*i*_). The feature vectors of residues in this window, *F*_*i*-*w *_... *F*_*i*+*w*_, are concatenated to produce the final vector representation of residue *x*_*i*_. If each residue has *d *features associated with it, the *w*mer encoding vector has length (2*w *+ 1) × *d *and is referred to as *w*mer(*F*_*i*_).

Figure 1 shows an example of the encoding process. Part (a) shows the PSSM for a sequence of interest with the central residue, Y, circled. Part (b) illustrates the *w*mer associated with the central residue for *w *= 3. Part (c) gives the arrangement of the feature vectors in the final vector encoding of the residue. The transformation in part (d) is explained in the next section.

### Kernel Functions

*svm*PRAT implements several kernel functions to capture similarity between pairs of *w*mers. Selection of an appropriate kernel function for a problem is key to the effectiveness of support vector machine learning.

#### Linear Kernels

Given a pair of *w*mers, *w*mer(*x*_*i*_) and *w*mer(*y*_*j*_) a linear kernel function can be defined between their feature matrices *w*mer(*F*_*i*_) and *w*mer(*G*_*j*_), respectively as(1)

where ⟨·,·⟩ denotes the dot-product operation between two vectors.

Some problems may require only approximate information for residue neighbors that are far away from the central residue while nearby residue neighbors are more important. For example, the secondary structure state of a residue is in general more dependent on the nearby sequence positions than the positions that are further away [28]. *svm*PRAT allows a window encoding shown in Figure 1(d) where the positions away from the central residue are averaged to provide a coarser representation while the positions closer to the central residue provide a finer representation. This two-parameter linear window kernel is denoted  and computes the similarity between features *w*mer(*F*_*i*_) and *w*mer(*G*_*j*_) as(2)

The parameter *w *governs the size of the *w*mer considered in computing the kernel. Rows within *i *± *f *contribute an individual dot product to the total similarity while rows outside this range provide only aggregate information. In all cases, *f *is less than or equal to *w *and as *f *approaches *w*, the window kernel becomes a sum of the dot products. This is the most fine-grained similarity measure considered and is equivalent to the one-parameter dot product kernel that equally weighs all positions of the *w*mer given by Equation 1. Thus, the two kernels  are  equivalent. Specifying *f *to be less than *w *merges neighbors distant from the central residue into only a coarse contribution to the overall similarity. For *f *<*w*, distant sequence neighbors are represented by only compositional information rather than specific positions where their features occur.

#### Exponential Kernels

*svm*PRAT implements the standard radial basis kernel function (*rbf*), defined for some parameter *γ *by(3)

*svm*PRAT also implements the normalized second order exponential (*soe*) kernel function shown to better capture pairwise information and improve accuracy for the secondary structure and local structure prediction problems [9,19]. Given any base kernel function , we define  as(4)

which is a second-order kernel in that it computes pairwise interactions between the elements *x *and *y*. We then define  as(5)

which normalizes  and embeds it into an exponential space.

By setting a specific *γ *parameter value and using normalized unit length vectors in Equation 3 it can be shown that the standard *rbf *kernel is equivalent (up to a scaling factor) to a first order exponential kernel which is obtained by replacing (*x, y*) with only the first-order term as (*x, y*) in Equation 4, and plugging this modified (*x, y*) in the normalization framework of Equation 5.

### Integrating Information

When multiple information in the form of different feature matrices is provided to *svm*PRAT, the kernel functions and information encoding per residue for each of the feature matrices remains the same. The final kernel fusion is accomplished using a weighted linear combination across the original base kernels. The weights for feature matrices can be set by the user.

For example, we can use the fusion of second-order exponential kernels on different features of a protein sequence. Considering two sequences with *k *sets of feature matrices *F*^*l *^and *G*^*l *^for *l *= 1,..., *k*, our fusion kernel is defined as(6)

where the weights *ω*_*l *_are supplied by the user. In most cases, these weights can be set to be equal but should be altered according to domain-specific information.

### Cascaded Models

Several prediction algorithms like PHD [17], PSIPRED [11] and YASSPP [9] developed for secondary structure prediction use a two-level cascaded prediction framework. This two-level framework trains two models, referred as the *L*_1 _and *L*_2 _models, which are connected together in a cascaded fashion. Both the *L*_1 _and *L*_2 _models train *K *one-versus-rest binary classification models for predicting a discrete label or a single ϵ-SVR regression model for estimating a continuous value. The predictions from the first-level *L*_1 _model are used as an input feature matrix along with the original features for training a *L*_2 _model [9]. Such cascaded predictions can be accomplished within *svm*PRAT's framework in the following way. First, the entire training set is used to train a *L*_1 _classification/regression model using the original input features. This is followed with a *n*-fold cross-validation step to generate predictions for the entire training set using the fold specific trained *L*_1 _model. In each iteration, 1/*n*-th of the dataset is set aside for prediction whereas the remainder of the dataset is used for training. The predictions from the *L*_1 _model are then used as a new input feature along with the original features to train a *L*_2 _model. The user may specify any desired weighting between original features and the *L*_1 _model predictions according to Equation 6. The final result is a cascaded prediction.

### Efficient Implementation

The runtime performance of *svm*PRAT is tied to the speed of computing the kernel function values between pairs of *w*mers. All the implemented kernel functions have to compute a dot product between the vector representations.

*svm*PRAT optimizes the computation time for the dot product based kernel functions given by Equation 2 by using the optimized CBLAS (Basic Linear Algebra Subprograms) routines that are a part of the ATLAS library project [20]. The CBLAS routines provide the standard building blocks for performing vector-based and matrix-based computations. In particular, the efficient vector operations available through CBLAS are used within *svm*PRAT's kernel function implementations. This allows *svm*PRAT to train models and generate predictions for test cases quickly.

We ported the CBLAS routines to all the architectures on which *svm*PRAT was complied and provide binaries compiled with and without the CBLAS routines (see the Availability Section).

### Predictions Output

For classification problems, *svm*PRAT's prediction program produces two outputs in text files. For every residue, raw prediction scores from the one-versus-rest SVMs are reported. In addition, each residue is assigned a class based on the maximum prediction score of the models. For regression problems, the output is a text file containing the estimated value produced by the ϵ-SVR model.

### Model Selection

*svm*PRAT provides an evaluation program called *svm*PRAT-E that allows the practitioner to determine the best set of parameters for a particular prediction problem using cross validation. For ease of use, a simple PERL script is provided which invokes *svm*PRAT-E for a fixed set of parameters to determine the best kernel and window lengths.

## Results

*svm*PRAT has been used in two previous experimental settings with success. TOPTMH is a transmembrane-helix segment identification and orientation system which utilizes *svm*PRAT[22]. It has achieved the best performance on a static independent benchmark [23]. The work by Kauffman et al. used *svm*PRAT to predict the ligand-binding residues of a protein [24]. This was shown to improve the quality of homology models of the protein's binding site.

In this work, we illustrate the capabilities of *svm*PRAT on a wide range of prediction problems. These case studies illustrate the effectiveness and generality of the software for sequence annotation problems. Problems involving disordered regions, DNA-protein interaction sites, residue contact order, and general local structure class are covered in the subsequent sections. Table 1 shows some characteristics of the datasets used in each problem and the reference work from which the data was derived.

**Table 1:** Problem-specific Datasets.

Problem	Source	Type	#C	#Seq	#Res	#CV	%
Disorder Prediction	DisPro [7]	Binary	2	723	215612	10	30
Protein-DNA Site	DISIS [6]	Binary	2	693	127240	3	20
Residue-wise Contact	SVM [15]	Regression	∞	680	120421	15	40
Local Structure	Profnet [35]	Multiclass	16	1600	286238	3	40

#C, #Seq, #Res, #CV, and % denote the number of classes, sequences, residues, number of cross validation folds, and the maximum pairwise sequence identity between the sequences, respectively. 8 represents the regression problem.

### Disorder Prediction

Some proteins contain regions which are intrinsically disordered in that their backbone shape may vary greatly over time and external conditions. A disordered region of a protein may have multiple binding partners and hence can take part in multiple biochemical processes in the cell which make them critical in performing various functions [29]. Disorder prediction is an example of a binary classification problem for sequence data. Disordered region prediction methods like IUPred [30], Poodle [18], and DISPro [7] make predictions using physiochemical properties of the amino acids or evolutionary information within a machine learning tool like bi-recurrent neural networks or SVMs.

*svm*PRAT was used to discriminate between residues belonging to ordered versus disordered regions. We assessed the value of several feature sets on this problem as an illustration of how *svm*PRAT may combine sequence information. The feature sets were PSI-BLAST PSSMS (), BLOSUM62 sequence features (), and predicted secondary structure (ℬ). See the Material Section for explanation of the different input features. The parameters *w *and *f *of the base window kernel () were varied to demonstrate their effects on prediction performance. Finally, linear (*lin*), radial basis function (*rbf*), and second order exponential (*soe*) kernels were all used to show how the similarity computation in  may be further processed to improve performance.

Table 2 shows the classification performance of various *svm*PRAT models on the disorder prediction problem. To notate the models, we use features as the main level text and kernel as the superscript (e.g.  uses PSSMs and secondary structure in the second order exponential kernel). *ROC *and *F*_1 _scores are reported for ten-fold cross validation which was the experimental protocol used to benchmark the DISPro [7]. Comparing the *ROC *performance of the , , and  models across different values of *w *and *f*, we observe that the *soe *kernel shows superior performance to the *lin *kernel and slightly better performance compared to the normalized *rbf *kernel used in this study. This is in agreement with the results of our previous studies for predicting secondary structure [9] and predicting RMSD between subsequence pairs [19] where the *soe *kernel outperformed the *rbf *kernel.

**Table 2:** Classification Performance on the Disorder Dataset.

	*w*	*f *= 1		*f *= 3		*f *= 5		*f *= 7		*f *= 9		*f *= 11	
		**ROC**	**F1**	**ROC**	**F1**	**ROC**	**F1**	**ROC**	**F1**	**ROC**	**F1**	**ROC**	**F1**

	3	0.775	0.312	**0.800**	0.350	-	-	-	-	-	-	-	-
	7	0.815	0.366	**0.817**	0.380	0.816	0.384	0.816	0.383	-	-	-	-
	11	0.821	0.378	0.826	0.391	**0.828**	0.396	0.826	0.400	0.824	0.404	0.823	0.403
	13	0.823	0.384	0.829	0.398	0.832*	0.405	0.830	0.404	0.828	0.407	0.826	0.409

	3	**0.811**	0.370	0.811	0.369	-	-	-	-	-	-	-	-
	7	0.845	0.442	**0.849**	0.450	0.848	0.445	0.845	0.442	-	-	-	-
	11	0.848	0.464	0.855	0.478	0.858	0.482	**0.858**	0.480	0.855	0.470	0.853	0.468
	13	0.848	0.473	0.855	0.484	0.859	0.490	0.861*	0.492	0.860	0.487	0.857	0.478

	3	0.815	0.377	**0.816**	0.379	-	-	-	-	-	-	-	-
	7	0.847	0.446	**0.852**	0.461	0.852	0.454	0.851	0.454	-	-	-	-
	11	0.848	0.469	0.856	0.482	0.860	0.491	**0.862**	0.491	0.861	0.485	0.862	0.485
	13	0.847	0.473	0.856	0.485	0.861	0.491	0.864	0.495	0.865*	0.494	0.864	0.492

	3	0.836	0.418	**0.838**	0.423	-	-	-	-	-	-	-	-
	7	0.860	0.472	**0.862**	0.476	0.860	0.473	0.859	0.468	-	-	-	-
	11	0.861	0.490	0.867	0.496	**0.868**	0.498	0.868	0.495	0.866	0.488	0.865	0.485
	13	0.860	0.497	0.867	0.503	0.870	0.503	0.871*	0.503	0.870	0.498	0.868	0.492

	3	**0.842**	0.428	0.841	0.428	-	-	-	-	-	-	-	-
	7	0.869	0.497	**0.870**	0.499	0.869	0.494	0.867	0.489	-	-	-	-
	11	0.871	0.516	0.875	0.518	**0.877**	0.517	0.877	0.512	0.874	0.508	0.873	0.507
	13	0.869	0.519	0.875	0.522	0.878	0.521	0.879**	0.519	0.879	0.518	0.876	0.514

DISPro [7] reports a *ROC *score of 0.878. The numbers in bold show the best models for a fixed *w *parameter, as measured by *ROC*. , ℬ, and  represent the PSI-BLAST profile, BLOSUM62, and YASSPP scoring matrices, respectively. *soe*, *rbf*, and *lin *represent the three different kernels studied using the *W*_*w*, *f *_as the base kernel. * denotes the best classification results in the sub-tables, and ** denotes the best classification results achieved on this dataset. For the best model we report a *Q*_2 _accuracy of 84.60% with an *se *rate of 0.33.

The performance of *svm*PRAT on the disorder prediction problem improved by using the , ℬ, and  feature matrices in combination rather than individually. Table 2 shows results for the successive use of , , and  features in the *soe *kernel: the additional features tend to improve performance. The flexible encoding introduced by *svm*PRAT shows some merit for the disorder prediction problem. The best performing fusion kernel shows comparable performance to DisPro [7] that encapsulates profile, secondary structure and relative solvent accessibility information within a bi-recurrent neural network.

#### Runtime Performance of Optimized Kernels

We benchmarked the learning phase of *svm*PRAT on the disordered dataset comparing the runtime performance of the program compiled with and without the CBLAS subroutines. These results are reported in Table 3 and were computed on a 64-bit Intel Xeon CPU 2.33 GHz processor for the , , and  kernels varying the *w*mer size from 11 to 15. Table 3 also shows the number of kernel evaluations for the different models. Using CBLAS, speedups ranging from 1.7 to 2.3 are achieved for disorder prediction. Similar speedups were noted for other prediction problems. *Disorder Prediction at CASP8*: CASP is a biennial protein structure prediction competition which includes a disorder prediction category (Competition Website: http://predictioncenter.org). We submitted predictions of disordered residues to the CASP8, the latest iteration of the competition. Our MARINER server (group 450) used *svm*PRAT as the backend prediction tool. CASP8 featured 125 target proteins with 27,775 residues out of which 11.2% were disordered residues.

**Table 3:** Runtime Performance of *svm*PRAT on the Disorder Dataset (in seconds).

	w = f = 11				w = f = 13				w = f = 15			
	**#KER**	**NO**	**YES**	**SP**	**#KER**	**NO**	**YES**	**SP**	**#KER**	**NO**	**YES**	**SP**
	1.93e+10	83993	45025	1.86	1.92e+10	95098	53377	1.78	1.91e+10	106565	54994	1.93
	1.91e+10	79623	36933	2.15	1.88e+10	90715	39237	2.31	1.87e+10	91809	39368	2.33
	2.01e+10	99501	56894	1.75	2.05e+10	112863	65035	1.73	2.04e+10	125563	69919	1.75

The runtime performance of *svm*PRAT was benchmarked for learning a classification model on a 64-bit Intel Xeon CPU 2.33 GHz processor. #KER denotes the number of kernel evaluations for training the SVM model. NO denotes runtime in seconds when the CBLAS library was not used, YES denotes the runtime in seconds when the CBLAS library was used, and SP denotes the speedup achieved using the CBLAS library.

The *svm*PRAT model employed for CASP8 was trained using profile information embedded within the *soe *kernel with a *w*mer size of 9. Table 4 gives the top performers from the disorder prediction category of CASP8. *svm*PRAT showed encouraging results compared to methods that are fine-tuned for disorder prediction. The blind evaluation done in CASP8 proves the ability of *svm*PRAT to adapt readily to different prediction problems.

**Table 4:** Disorder Prediction Performance at CASP8.

Method	ROC	Q_2	*S*_*w*_
MULTICOM	0.92	0.81	0.61
CBRC-DP_DR	0.91	0.81	0.62
GS-MetaServer2	0.91	0.83	0.66
McGuffin	0.91	0.82	0.64
DISOclust	0.91	0.82	0.64
GeneSilicoMeta	0.90	0.83	0.655
Poodle	0.90	0.80	0.61
CaspIta	0.89	0.78	0.571
fais-server	0.89	0.78	0.56
MULTICOM-CMFR	0.89	0.82	0.64
MARINER*	0.88	0.80	0.61

* - MARINER used *svm*PRAT to train models for disorder prediction in participation at CASP8 using the  kernel with *w *= *f *= 11. We used the 723 sequences with disordered residues from the DisPro [7] dataset. The results are the official results from the CASP organizers and were presented by Dr. Joel Sussman at the Weizmann Institute of Science. *Q*_2 _denotes the 2-state accuracy for the prediction and *S*_*w *_is a weighted accuracy rewarding the prediction of disordered residue.

The results from the CASP assessors were published recently [31] and show that the top performers based on a weighted accuracy are consensus-based methods. Poodle is a SVM-based approach that uses two sets of cascaded classifiers trained separately for long and short disordered regions. *svm*PRAT can easily train two separate cascaded models for long and short disordered regions and thus incorporate the domain insight introduced by Poodle, in an efficient and quick manner.

### Contact Order Prediction

Pairs of residues are considered to be in contact if their *C*_*β *_atoms are within a threshold radius, generally 12 Å. Residue-wise contact order [15] is defined as the average distance separation between contacting residues within a sphere of set threshold. Contact order prediction is an example of a regression problem for sequence data: the value to be predicted is a positive integer rather than a class. To predict contact order, Song and Burage [15] used support vector regression with a variety of sequence features including PSI-BLAST profiles, predicted secondary structure from PSIPRED [11], amino acid composition, and molecular weight. Critical random networks have also been applied to solve the problem [16]. We used *svm*PRAT to train ϵ-SVR regression models for residue-wise contact order estimation. PSSM and predicted secondary structure,  and  respectively, were used as features in the *soe *kernel. The window kernel parameters *w *and *f *were varied again to study their impact. Evaluation was carried out using 15-fold cross validation on the dataset of Song and Burage [15].

Table 5 shows the average per protein correlation coefficient and RMSE values of *svm*PRAT models. The best performing model used a fusion of  and  feature matrices and improves *CC *by 21% and *RMSE *by 17% over the ϵ-SVR technique of Song and Barrage [15]. Their method used the standard *rbf *kernel with similar local sequence-derived amino acid and predicted secondary structure features. The major improvement of our method can be attributed to our fusion-based kernel setting with efficient encoding and the normalization introduced in by the second order exponential kernel (Equation 5). For the window kernel parameters, we observe that models trained with *f *<*w *generally shows better *CC *and *RMSE *values for residue-wise contact order prediction.

**Table 5:** Residue-wise Contact Order Estimation Performance

	*w*	*f *= 1		*f *= 3		*f *= 5		*f *= 7		*f *= 9		*f *= 11	
		**CC**	**RMSE**	**CC**	**RMSE**	**CC**	**RMSE**	**CC**	**RMSE**	**CC**	**RMSE**	**CC**	**RMSE**

	3	0.704	0.696	**0.708**	0.692	-	-	-	-	-	-	-	-
	7	0.712	0.683	0.719	0.677	**0.723**	0.672	0.722	0.672	-	-	-	-
	11	0.711	0.681	0.720	0.673	**0.725**	0.667	0.725	0.666	0.724	0.666	0.722	0.667
	15	0.709	0.680	0.719	0.672	0.726**	0.665	0.726	0.664	0.725	0.664	0.723	0.664

*CC *and *RMSE *denotes the average correlation coefficient and RMSE values. The numbers in bold show the best models as measured by *CC *for a fixed *w *parameter. , and  represent the PSI-BLAST profile and YASSPP scoring matrices, respectively. *soe*, *rbf*, and *lin *represent the three different kernels studied using the  as the base kernel. * denotes the best regression results in the sub-tables, and ** denotes the best regression results achieved on this dataset. For the best results the *se *rate for the *CC *values is 0.003. The published results [15] uses the default rbf kernel to give *CC *= 0.600 and *RMSE *= 0.78.

### Protein-DNA Interaction Site Prediction

When it is known that the function of a protein is to bind to DNA, it is highly desirable from an experimental point of view to know which parts of the protein are involved in the binding process. Interaction is typically defined in terms of contacts between the protein and DNA in their co-crystallized structure: residues within a distance threshold of the DNA are considered interacting while the remaining residues are considered non-interacting. This is another example of a binary classification problem for sequence data. Several researchers have presented methods to identify DNA-binding residues. DISIS [6] uses support vector machines and a radial basis function kernel with PSSMs, predicted secondary structure, and predicted solvent accessibility as input features while Ahmad and Sarai employ a neural network method with PSSMs as input [32].

*svm*PRAT was used to train binary classification models on the DISIS dataset [6]. Following DISIS, we performed 3-fold cross validation on our models ensuring that the sequence identity between the different folds was less than 40%. During the experiments, we found that window kernels with *w *= *f *performed the best and therefore omit other values for the parameters.

Table 6 gives the performance of *svm*PRAT models on DNA interaction site prediction. The model obtained by combining the  and  features gives a raw *Q*_2 _accuracy of 83%. DISIS uses a two-level approach to solve this problem. The first level, which uses SVM learning with profiles, predicted secondary structure, and predicted solvent accessibility as inputs, gives *Q*_2 _= 83% to which our approach compares favorably. DISIS further smooths this initial prediction using a rule-based approach that improves accuracy. We have not yet explored this type of rule-based approach.

**Table 6:** Classification Performance on the Protein-DNA Interaction Site Prediction.

	*w *= *f *= 3		*w *= *f *= 7		*w *= *f *= 11	
	**ROC**	**F1**	**ROC**	**F1**	**ROC**	**F1**

	**0.756**	0.463	0.758*	0.469	0.748	0.452
	**0.753**	0.465	**0.754**	0.462	0.759	0.466
	**0.754**	0.466	**0.756**	0.468	0.763	0.468

The numbers in bold show the best models for a fixed *w *parameter, as measured by *ROC*. , and  represent the PSI-BLAST profile and YASSPP scoring matrices, respectively. *soe*, *rbf*, and *lin *represent the three different kernels studied using the  as the base kernel. * denotes the best classification results in the sub-tables, and ** denotes the best classification results achieved on this dataset. For the best model we report a *Q*_2 _accuracy of 83.0% with an *se *rate of 0.34.

### Local Structure Alphabet Prediction

The notion of local, recurring substructure in proteins has existed for many years primarily in the form of the secondary structure classifications. Many local structure alphabets have been generated by careful manual analysis of structures such as the DSSP alphabet [33]. More recently, local structure alphabets have been derived through pure computational means. One such example are the Protein Blocks of de Brevern et al. [13] which were constructed through the use of self-organizing maps. The method uses residue dihedral angles during clustering and attempts to account for order dependence between local structure elements which should improve predictability.

We chose to use the Protein Blocks [13] as our target alphabet as it was found to be one of the best local structure alphabets according to conservation and predictability [12]. There are sixteen members in this alphabet which significantly increases prediction difficulty over traditional three-state secondary structure prediction.

We used a dataset consisting of 1600 proteins derived from the SCOP database version 1.57, classes A to E [34]. This dataset was previously used for learning profile-profile alignment scoring functions using neural networks [35]. To compute the true annotations, we used the three-dimensional structures associated with the proteins to assign each residue one of the Protein Blocks.

We used a small subset of the 1600 proteins to tune the *w *and *f *windowing parameters with the *soe *kernel. We found *w *= *f *worked well on the subset and subsequently restricted the large-scale experiments to this case. Three-fold cross validation was done on all 1600 proteins for each parameter set and for both the *soe *and *rbf *kernels. Table 7 reports the classification accuracy in terms of the *Q*_16 _accuracy and average *ROC *scores for different members of the Protein Blocks.

**Table 7:** Classification Performance on the Local Structure Alphabet Dataset.

	*w *= *f *= 5		*w *= *f *= 7		*w *= *f *= 9	
	**ROC**	***Q*_16_**	**ROC**	***Q*_16_**	**ROC**	***Q*_16_**

	0.82	64.9	0.81	64.7	0.81	64.2
	0.83	67.3	0.82	67.7	0.82	67.7
	0.84	66.4	0.84	66.9	0.83	67.2
	0.85	68.0	0.84	68.5	0.83	68.9**

*w *= *f *gave the best results on testing on few sample points, and hence due to the expensive nature of this problem, we did not test it on a wide set of parameters. ** denotes the best scoring model based on the *Q*_16 _scores. For this best model the *se *rate of 0.21.

From Table 7 we see that the *soe *kernel provides a small performance boost over the *rbf *kernel. The addition of predicted secondary structure information from YASSPP ( features) improves the *Q*_16 _performance as would be expected for local structure prediction. Our *Q*_16 _results are very encouraging, since they are approximately 67%, whereas the prediction accuracy for a random predictor would be 6.25% only. Competitive methods for predicting Protein Blocks from sequence reported a *Q*_16 _accuracy of 40.7% in [36] and 57.9% in [12].

### Datasets

Our empirical evaluations are performed for different sequence annotation problems on previously defined datasets. Table 1 presents information regarding the source and key features of different datasets used in our cross validation and comparative studies. We ensured that the pairwise sequence identities for the different datasets was less than 40%.

We utilized cross validation as our primary evaluation protocol. In *n*-fold cross validation, data are split into *n *sets. One of the *n *sets is left out while the others are used to train a model. The left out data are then predicted and the performance is noted. This process repeats with a different set left out until all *n *sets have been left out once. The average performance over all *n*-folds is reported. Where possible, we used the same splits of data as have been used in previous studies to improve the comparability of our results to earlier work.

### Evaluation Metrics

We measure the quality of the classification methods using the receiver operating characteristic (*ROC*) scores. The *ROC *score is the area under the curve that plots the fraction of true positives against the fraction of false positives for different classification thresholds [37]. In all experiments, the *ROC *score reported is averaged over the *n *folds of cross validation. When the number of classes is larger than 2, we use a one versus rest *ROC *scores and report the average across all classes.

We also compute other standard statistics defined in terms of the number of true positives (TP), false positives (FP), true negatives (TN), and false negatives (FN). These standard statistics are the following:(7)

For *K*-way classification, performance is summarized by *Q*_*K*_, defined as(10)

where *N *is the total number residues and *TP*_*i *_is the number of true positives for class *i*.

The *ROC *score serves as a good quality measure in the case of unbalanced class sizes where *Q*_*K *_may be high simply by predicting the most frequent class. This is often true for binary classification problems with very few positive examples. In such cases, it is essential to observe the precision and recall values which penalize the classifiers for under-prediction as well as over-prediction. The *F*_1 _score is a weighted average of precision and recall lying between 0 and 1, and is a good performance measure for different classification problems.

Regression performance is assessed by the Pearson correlation coefficient (*CC*) and the root mean square error (*RMSE*) between the predicted and observed true values for every protein in the datasets. The *CC *statistic ranges from -1 to +1 with larger values being better while *RMSE *is larger than zero with lower values implying better predictions. The results reported are averaged across the different proteins and cross validation folds.

For the best performing models, we also report the standard error, *se*, of *Q*_*K *_and *CC *scores, defined as(11)

where  is the sample standard deviation and *N *the number of data points. This statistic helps assess how much performance varies between proteins.

### Input Information

#### Position Specific Scoring Matrices

For a sequence of length *n*, PSI-BLAST [38] generates a position-specific scoring matrix (PSSM) referred to as . The dimensionality of  is *n *× 20, where the 20 columns of the matrix correspond to the twenty amino acids. The profiles in this study were generated using the version of the PSI-BLAST available in NCBI's 2.2.10 release of the BLAST package. PSI-BLAST was run as blastpgp -j 5 -e 0.01 -h 0.01 and searched against NCBI's NR database that was downloaded in November of 2004 (2,171,938 sequences).

#### Predicted Secondary Structure Information

We used the YASSPP secondary structure prediction server [9] with default parameters to generate the *S *feature matrix of dimensions *n *× 3. The (*i*, *j*)th entry of this matrix represents the propensity for residue *i *to be in state *j*, where *j *∈ {1, 2, 3} corresponds to the three secondary structure elements: alpha helices, beta sheets, and coil regions.

#### Position Independent Scoring Matrices

Position independent sequence features were created for each residue by copying the residue's corresponding row of the BLOSUM62 scoring matrix. This resulted in a *n *× 20 feature matrix referred to as ℬ.

By using both PSSM and BLOSUM62 information, a SVM learner can construct a model that is based on both position independent and position specific information. Such a model is more robust to cases where PSI-BLAST could not generate correct alignments due to lack of homology to sequences in the NR database.

## Conclusions

In this work we have presented a general purpose support vector machine toolkit that builds protein sequence annotation models. Dubbed *svm*PRAT, the toolkit's versatility was illustrated by testing it on several types of annotations problems. These included binary classification to identify transmembrane helices and DNA-interacting residues, *K*-way classification to identify local structural class, and continuous predictions to estimate the residue-wise contact order. During our evaluation, we showed the ability of *svm*PRAT to utilize arbitrary sequence features such as PSI-BLAST profiles, BLOSUM62 profiles, and predicted secondary structure which may be used with several kernel functions. Finally *svm*PRAT allows the incorporation of of local information at different levels of granularity through its windowing parameters. Our experiments showed that this allows it to achieve better performance on some problems. *svm*PRAT's key features include: (i) implementation of standard kernel functions along with powerful second-order exponential kernel, (ii) use of any type of sequence information associated with residues for annotation, (iii) flexible window-based encoding scheme, (iv) optimized for speed using fast solvers, (v) capability to learn two-level cascaded models, and (vi) available as pre-compiled binaries for various architectures and environments.

We believe that *svm*PRAT provides practitioners with an efficient and easy-to-use tool for a wide variety of annotation problems. The results of some of these predictions can be used to assist in solving the overarching 3D structure prediction problem. In the future, we intend to use this annotation framework to predict various 1D features of a protein and effectively integrate them to provide valuable supplementary information for determining the 3D structure of proteins.

## Availability and Requirements

• **Project Name**: *svm*PRAT

• **Website**: http://www.cs.gmu.edu/~mlbio/svmprat

• **Mirror**: http://bio.dtc.umn.edu/svmprat

• Operating Systems and Architectures: 64-bit Linux, 32-bit Linux, 64-bit MS Windows, 32-bit Darwin (Mac OSX), SUN Solaris Sun-Blade-1500-Solaris

• Programming Language: ANSI C

• Additional Features: Compiled with/without optimized CBLAS

• License: GNU GPL

• Restrictions for use by non-academics: Yes

### Web Interface

Even though *svm*PRAT is easy to use and is available across a wide variety of platforms and architectures, we also provide biologists the functionality to predict local structure and function predictions using our web server, MONSTER (Minnesota prOteiN Sequence annotaTion servER). *svm*PRAT serves as the backend for MONSTER and can be accessed easily via the web link http://bio.dtc.umn.edu/monster.

## Authors' contributions

HR developed the *svm*PRAT code. HR and CK performed the experimental evaluation. GK provided support and developed the SVM routines from the *SV M*_*light *_package. HR and GK developed MONSTER. All authors wrote the manuscript, read the manuscript, and approved for final submission.
